# Comparison of Serum and Cerebrospinal Fluid Neurofilament Light Chain Concentrations Measured by Ella™ and Lumipulse™ in Patients with Cognitive Impairment

**DOI:** 10.3390/diagnostics14212408

**Published:** 2024-10-29

**Authors:** Teresa Urbano, Riccardo Maramotti, Manuela Tondelli, Chiara Gallingani, Chiara Carbone, Najara Iacovino, Giulia Vinceti, Giovanna Zamboni, Annalisa Chiari, Roberta Bedin

**Affiliations:** 1 Neuroimmunology Laboratory, Department of Biomedical, Metabolic and Neural Sciences, University of Modena and Reggio Emilia, Baggiovara Hospital, 41126 Modena, Italy; teresa.urbano@unimore.it (T.U.); roberta.bedin@unimore.it (R.B.); 2 Environmental, Genetic and Nutritional Epidemiology Research Center (CREAGEN), Department of Biomedical, Metabolic and Neural Sciences, University of Modena and Reggio Emilia, 41125 Modena, Italy; 3 Department of Biomedical, Metabolic and Neural Sciences, University of Modena and Reggio Emilia, 41125 Modena, Italy; riccardo.maramotti@unimore.it (R.M.); gallinganichiara@gmail.com (C.G.); chiara.carbone@unimore.it (C.C.); najara.iacovino@unimore.it (N.I.); giovanna.zamboni@unimore.it (G.Z.); 4 Department of Physics, Informatics and Mathematics, University of Modena and Reggio Emilia, 41125 Modena, Italy; 5 Department of Mathematics and Computer Science, University of Ferrara, 44121 Ferrara, Italy; 6 Neurology Unit, Baggiovara Hospital, 41126 Modena, Italy; gvinceti@gmail.com (G.V.); chiari.annalisa@aou.mo.it (A.C.)

**Keywords:** assays, cerebrospinal fluid, dementia, neurofilament light chain protein, serum

## Abstract

Objective: Neurofilament light chain proteins (NfLs) are considered a promising biomarker of neuroaxonal damage in several neurological diseases. Their measurement in the serum and cerebrospinal fluid (CSF) of patients with dementia may be especially useful. Our aim was to compare the NfL measurement performance of two advanced technologies, specifically the Ella™ microfluidic platform and the Lumipulse™ fully automated system, in patients with cognitive disorders. Methods: Thirty subjects with neurodegenerative cognitive disorders (10 with Alzheimer’s Disease, 10 with Frontotemporal Dementia, and 10 with non-progressive Mild Cognitive Impairment) seen at the Cognitive Neurology Clinic of Modena University Hospital (Italy) underwent CSF and serum NfL measurement with both the Ella™ microfluidic platform (Bio-Techne, Minneapolis, MN, USA)) and the Lumipulse™ fully automated system for the CLEIA (Fujirebio Inc., Ghent, Belgium). Correlation and regression analyses were applied to assess the association between NfL concentrations obtained with the two assays in CSF and serum. The Passing–Bablok regression method was employed to evaluate the agreement between the assays. Results: There were high correlations between the two assays (r = 0.976, 95% CI. 0.950–0.989 for CSF vs. r = 0.923, 95% CI 0.842–0.964 for serum). A Passing–Bablok regression model was estimated to explain the relationship between the two assays, allowing us to switch from one to the other when only one assay was available. Conclusions: We found a good degree of correlation between the two methods in patients with neurocognitive disorders. We also established a method that will allow comparisons between results obtained with either technique, allowing for meta-analyses and larger sample sizes.

## 1. Introduction

Neurofilament light chain proteins (NfLs) are class IV intermediate filament proteins of 68 kDa and represent the most abundant intermediate filament protein expressed in mature neurons, providing structural stability and resistance to mechanical stress [1]. After neuronal and axonal damage, increased quantities of NfLs are released into the cerebrospinal fluid (CSF) and drained into the blood [2,3]. Among other classes of neurofilaments (heavy and medium chain), NfLs are considered one of the most promising biomarkers, reflecting neuroaxonal damage in a wide variety of neurological diseases [4,5]. Recent studies have also suggested that an altered permeability of the blood–brain barrier and blood–CSF barrier can influence blood NfL concentrations, although the exact influence of such barrier permeability remains to be elucidated [6,7].

In the past three decades, ultrasensitive immunoassay technologies have been developed with the purpose of making these biomarkers more clinically useful. With these improvements, NfLs have begun to be measured in blood, with studies reporting high correlations between serum and CSF levels [2,8,9]. The gold standard for NfL measurement in terms of sensitivity is the single-molecule array (SIMOA™) digital immunoassay technology from Quanterix. However, the assay cost often represents a limitation to its availability in clinical laboratories. A recently introduced and cheaper ultrasensitive assay based on a microfluidic enzyme-linked immunosorbent assay (ELISA) system is Ella™ from Bio-Techne [10,11,12]. Ella™ performs immunoassays in a microfluidic cartridge, measuring up to 72 samples in triplicate inside glass nanoreactors using a fluorescent substrate. Another very recent introduction is the assay based on the Lumipulse™ platform from Fujirebio Inc. It is a fully automated chemiluminescent enzyme immunoassay (CLEIA), allowing for fully automated processing of samples. The capturing antibody is combined with a detection antibody directly labelled with alkaline phosphatase [13].

As of today, only one study conducted in patients with multiple sclerosis directly compared the performance of the Ella™ and Lumipulse™ assays [11]. No studies have compared these assays in patients with cognitive impairment due to neurodegenerative dementias such as Alzheimer’s Disease (AD) and Frontotemporal Dementia (FTD). We performed a comparative analysis of NfL measurement in both CSF and serum samples from patients with cognitive disorders using two advanced technologies, specifically the Ella™ microfluidic platform and the Lumipulse™ fully automated system.

## 2. Materials and Methods

### 2.1. Study Population and Sample Collection

Thirty consecutive eligible subjects seen at the Cognitive Neurology Clinic of Modena University Hospital, Northern Italy, who had undergone blood testing and lumbar puncture as part of their diagnostic workup and had been clinically followed-up for two years, were recruited. They were included in the present study if they had one of the following clinical diagnoses: probable AD supported by abnormal CSF tau and amyloid biomarkers [14], FTD including its behavioural and aphasic variants [15,16], and non-progressive cognitive impairment. The latter diagnostic group included subjects that had initially received a clinical diagnosis of Mild Cognitive Impairment (MCI) [17] or Mild Neurocognitive Disorder [18] but were found to have normal CSF AD biomarkers and normal structural magnetic resonance imaging of the brain and remained clinically stable for at least 2 years. All individuals had provided written informed consent for the use of CSF and serum samples and personal data for both diagnostic and research purposes before sample collection (Ethics Committee approvals no 26/2017, 372/2021).

Clinical diagnoses were given by a team that included neurologists with expertise in cognitive disorders (AC, MT, GV, GZ) through neurological, neuroimaging, and neuropsychological assessments. For each patient, data on sex, age, and mini-mental state examination (MMSE) at the time of sampling were collected. Venepunctures and lumbar punctures were performed in the morning in fasting patients. Standard International Procedures for CSF and serum biobanking were followed [19], as previously described in detail [20,21,22]. Samples were immediately anonymized using an alphanumeric code upon their arrival at the neuro-immunology laboratory, located at the same University Hospital, and processed within 30 min. After centrifugation at 2500× *g* for 10 min at controlled room temperature, the sample supernatant was aliquoted into polypropylene sterile vials and kept frozen at −80 °C until analysis. Before testing, samples were thawed and centrifuged at 2500× *g* for 5 min in strict accordance with the manufacturer’s protocols.

### 2.2. NfL Assays

CSF and serum NfL concentrations were assessed using two platforms: the commercial Ella™ microfluidic platform (Bio-Techne, Minneapolis, USA) and the Lumipulse™ fully automated system for the CLEIA (Fujirebio Inc., Ghent, Belgium). The Ella™ device utilized the Human NF-L Simple Plex cartridge-based assay (ProteinSimple, San Jose, CA, USA), following the manufacturer’s instructions. The Lumipulse™ G600II fully automated instrument employed Lumipulse G NfL CSF and Lumipulse G NfL Blood Chemiluminescent Enzyme ImmunoAssays. Ella™ was considered the reference method, given its previous utilization, validation, and standardization in our neurology clinic [23,24]. The main characteristics of the two assays are reported in Table S1. Serum and CSF dilutions were manually performed for Ella™ according to the strictly recommended procedures of the manufacturer.

### 2.3. Statistical Analysis

Statistical analyses were performed using Stata statistical software (Stata 18.0-SE, StataCorp LLC, College Station, TX, USA, 2023) and MATLAB version R2022b [25]. The Shapiro–Wilk test was used to check the normality of the distribution of NfL concentrations in CSF and serum for both assays. As none of the variables were found to be normally distributed, Spearman correlations were used to test the association between serum and CSF NfL concentrations in the same sample series.

Crude and adjusted models were used to perform linear and spline regression analyses to assess the association between NfL concentrations in CSF and serum with the two assays. In the adjusted model, age was used as a continuous adjustment variable. Beta linear regression coefficients (β), with their 95% confidence intervals (CIs), were computed for each regression analysis to compare the effect strength of each independent variable (NfL levels measured with Ella™) on the dependent variable (NfL levels measured with Lumipulse™). To assess the potential non-linearity of our associations, we used regression analyses fitted on a restricted cubic spline model using three knots at fixed percentiles (10th, 50th, and 90th) previously implemented [26,27]. Linear and spline fits were then compared using ANOVA. The Passing–Bablok regression method was also employed to evaluate agreement between assays.

## 3. Results

**Figure 1:**
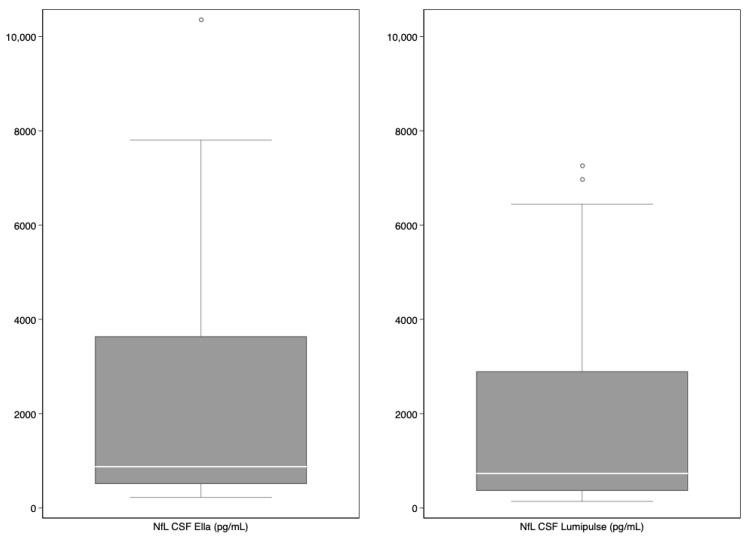

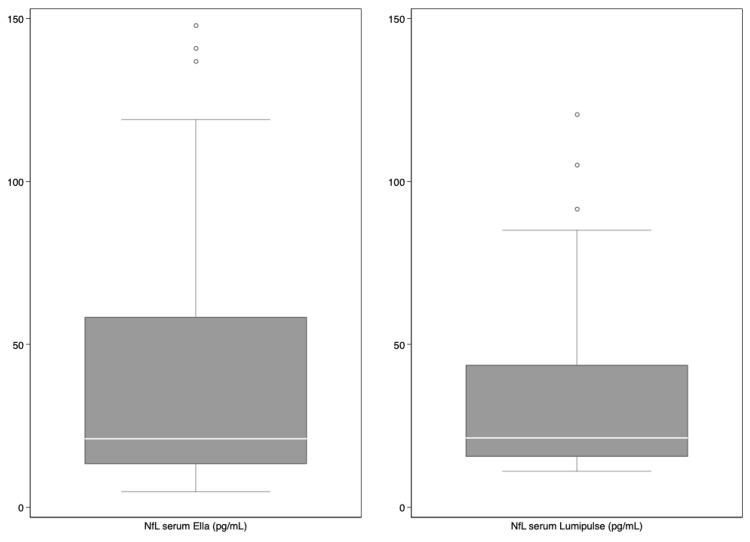
Boxplots of median neurofilament light chain (NfL) (in pg/mL) concentrations in cerebrospinal fluid (CSF) and serum using the two assays (Ella™ and Lumipulse™).

The characteristics of the subjects included in the study are reported in Table 1. The median concentrations of CSF and serum NfL were, respectively, 879 pg/mL (interquartile range (IQR) 510–3641 pg/mL) and 21.00 pg/mL (13.30–58.40 pg/mL) with Ella™ and 735.50 pg/mL (IQR 365–2897 pg/mL) and 21.26 pg/mL (15.54–43.69 pg/mL) with Lumipulse™ (Table 1 and Figure 1).

The median NfL levels were found to be higher in males compared to females, with the NfL serum levels measured with Ella™ showing the weakest difference between sexes (Table S2). As for the diagnostic group, NfL concentrations measured through the two assays in both blood and CSF were higher in FTD compared to AD and non-progressive MCI, as well in AD compared to non-progressive MCI.

The results of the Spearman correlations are reported in Table 2, showing a high level of correlation between the two assays. The CSF NfL concentration demonstrated a slightly stronger correlation compared to that in serum.

The variance explained by the linear regression models was high for both serum and CSF (adjusted R2 = 0.908 for CSF vs. adjusted R2 = 0.885 for serum). According to the results of the models, both the serum and CSF levels measured with the two assays were linearly dependent (β: 0.77, 95% CI 0.68–0.86 for CSF vs. β: 0.64, 95% CI 0.55–0.73 for serum). Table S3 reports the crude and adjusted-by-age linear regression models explaining the relationship between the two assays.

**Figure 2:**
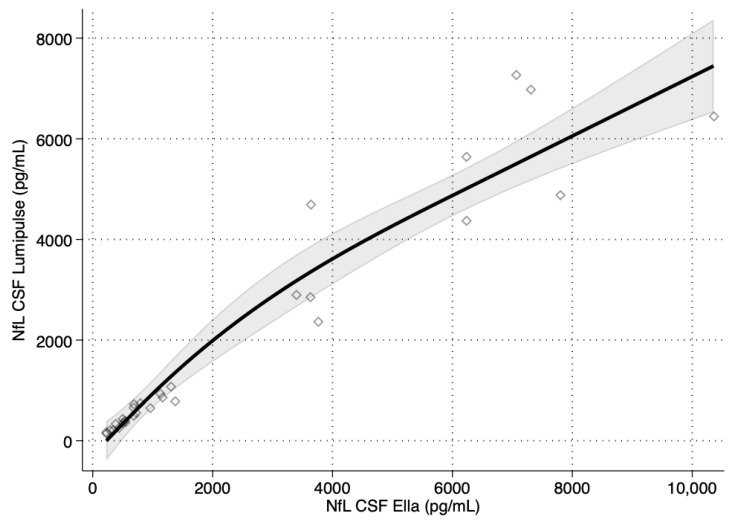

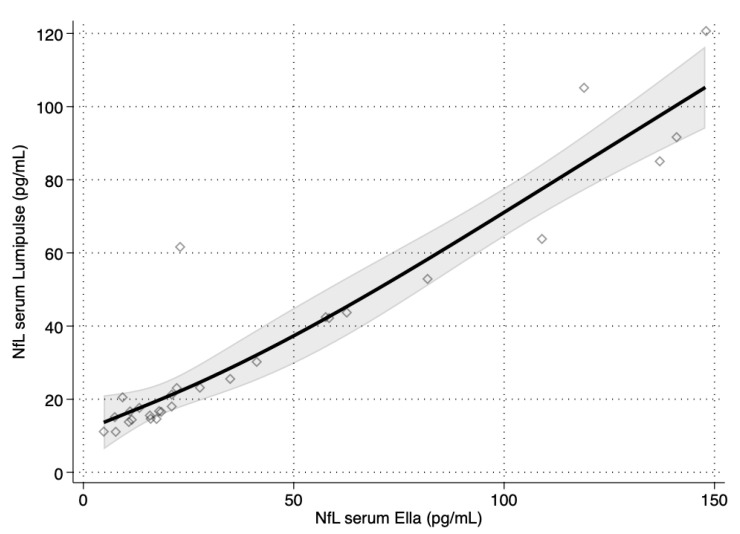
Spline regression analysis of neurofilament light chain (NfL) concentrations using the two assays (Ella™ and Lumipulse™). The solid line represents the regression analysis with upper and lower confidence interval limits (shaded grey area). Diamonds represent individual observations.

The spline regression analysis using Ella™ as a reference method (independent variable) also explained the high variance (adjusted R2 = 0.920 for CSF vs. adjusted R2 = 0.881 for serum). However, the ANOVA showed no statistically significant difference between the linear and spline models (*p* = 0.096 for CSF vs. *p* = 0.567 for serum), indicating that the two assays were almost linearly dependent (Figure 2).

The relationship among variables was also examined using a scatterplot matrix and showed a good correlation between NfL CSF and serum levels in both assays (Figure S1).

Given the non-normal distribution of data, we used the non-parametric Passing–Bablok regression model to estimate a formula that explains the relationship between the two assays, allowing for the estimation of the values with one method when only the other is available and vice versa. When only Ella™ is available, the formulae used to obtain the corresponding value in Lumipulse™ (y) in relation to Ella™ (x) are

y(Lumipulse™) = 0.8261 × x(Ella™) − 45.38 (for CSF)

y(Lumipulse™) = 0.5775 × x(Ella™) + 7.543 (for serum)

On the other hand, when only Lumipulse™ (y) is available, the formulae used to obtain the corresponding Ella™ (x) value are

x(Ella™) = 1.211 × y(Lumipulse™) + 45.38 (for CSF)

x(Ella™) = 1.732 × y(Lumipulse™) − 7.543 (for serum)

**Figure 3:**
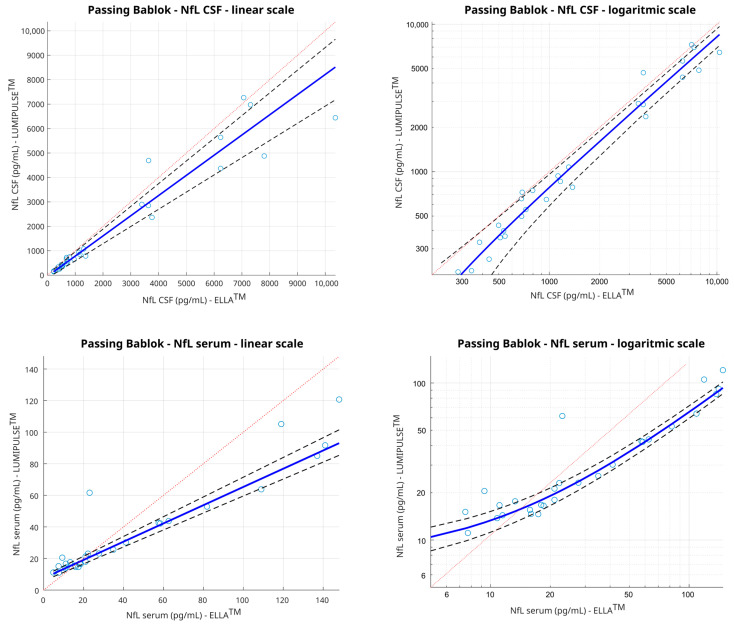
Passing–Bablok regression analysis of neurofilament light chain (NfL) concentration calculated with 30 samples by the Ella™ platform compared to the Lumipulse™ platform. Results are shown both on linear and logarithmic scales. Solid blue line: Passing–Bablok regression line; dashed red line: identity line; dashed grey lines: 95% confidence intervals. These graphics may be interpreted as follows: if the red identity line is outside of the credible intervals of the Passing–Bablok regression line, this indicates a proportional difference between the two assays and thus a need for correction.

As for the equations, the slope of the Passing–Bablok regression line is reported in Table 3. The 95% CI of the slope does not include 1, which indicates a significant proportional difference between the two methods. Consequently, the application of a correction coefficient is necessary (Table 3 and Figure 3).

## 4. Discussion

The quantification of NfL concentrations in CSF and serum is gaining increasing importance in the diagnosis of several neurological conditions and is especially promising in neurodegenerative disorders associated with dementia [28,29,30,31,32]. In this study, we compared CSF and serum NfL levels in patients with cognitive disorders due to different diseases (AD, FTD, and non-progressive MCI), measuring them with two different assays: Lumipulse™ and Ella™. We demonstrated that, albeit highly correlated, the two methods show a significant proportional difference. We therefore estimated for the first time a formula allowing for the conversion from one to the other assay.

To the best of our knowledge, no previous similar comparisons have been made in patients with cognitive impairment or neurodegenerative diseases, as only one study to date has reported comparisons of NfL levels with Ella™ and Lumipulse™ in patients with multiple sclerosis [11]. Several studies, instead, have compared Ella™ with SIMOA™ technology. In these studies, the differences between the two assays were ascribed to the use of different calibrators, namely naturally derived bovine NfLs for Ella™ and recombinant human NfLs for SIMOA™ [33]. In our study, this should not be an issue as Lumipulse™, like Ella™, uses a naturally derived bovine NfL calibrator.

Globally, the results obtained showed a very high level of correlation with the calculated correlation coefficients of 0.976 and 0.923 for CSF and serum, respectively. These findings confirm a good degree of correlation between different assays, similarly to what was reported in studies comparing Ella™ to SIMOA™ assays in patients with multiple sclerosis and dementia [12,33]. To date, no studies in patients with dementia or cognitive impairment have used the Lumipulse™ instrument to measure NfL concentrations, as they have only used Ella™, SIMOA™, or manual ELISA techniques [34,35,36,37,38]. The good degree of correlation between the two methods and the estimation of conversion formulae will allow for comparisons of results obtained with either method. Importantly, it will also allow for the merging of datasets in which NfLs were measured with either technique with the goal of obtaining larger population samples and performing comparisons and meta-analyses.

We observed that the CSF concentrations exceeded those in the serum with both the assays, being 42- and 35-fold higher using Lumipulse™ and ELLA™, respectively. This is in line with previous studies, which have consistently shown that serum NfLs are a reliable proxy for CSF NfL concentrations in different neurological diseases, albeit measurable in smaller concentrations [24,39,40,41].

We found that the measurements of NfL levels obtained with the two assays were more strongly correlated in the CSF compared to the serum in our cohort of patients with cognitive impairment. This is in line with the findings of a recent study comparing the two assays in patients with multiple sclerosis [11] and may be explained by the fact that several factors that affect NfL levels in the serum, such as creatinine and cholesterol levels, do not pass the blood–CSF barrier and thus do not influence NfL levels in the CSF [42,43].

Besides pathological conditions, it is well known that the levels of NfLs in both CSF and serum increase with age [44,45]. We found that the adjustment for age did not substantially change the linear relationship between the measurements obtained with the two different assays; thus, the conversion formula from one to the other assay that we estimated can be used independently of age for people in the investigated age spectrum.

Our study has limitations. First, the size of our dataset is limited to 30 subjects. However, the univariate analysis previously described is also applicable in the context of a limitless sample size, and the narrow width of the confidence intervals suggests the stability of the regression models across subjects. Moreover, our results will inherently allow us to move towards larger datasets obtained from combining smaller datasets. Moreover, we cannot ignore that patients with stable MCI for at least 2 years could possibly develop dementia after the end of the follow-up; nevertheless, even considering this possibility, the good correlation between the two methods and the estimation of the conversion formula remains valid.

Our study also has strengths: we purposefully included a sample of patients with the two most common neurodegenerative dementias (AD and FTD) as well as a sample of subjects with cognitive concerns without any biomarker abnormality or clinical evidence of progression, allowing us to verify the correspondence between the two different methodologies for NfL measurement across a spectrum of patients representative of those usually presenting in cognitive neurology clinics, for whom the measurement of NfLs will likely become part of the routine diagnostic pathway in the near future.

## 5. Conclusions

In conclusion, the quantification of NfLs is gaining an increasingly important role in neurodegenerative diseases associated with dementia; thus, it is essential to know all the possible sources of variation in their measurement, including the type of assay. NfL dosing in dementia research undoubtedly offers a promising avenue for advancing early detection and differential diagnosis between different types of dementia and for monitoring therapeutic interventions. By establishing a reliable conversion factor, the results of different studies can be fully compared, even when values are obtained with different methods in different biological fluids. In this context, it is important to enhance our understanding of the clinical significance of NfLs, which will, in turn, promote the wider adoption of this promising biomarker.

## Figures and Tables

**Table 1:** Median and interquartile range (IQR) concentrations of age, mini-mental state examination (MMSE) score, and neurofilament light chain (NfL) according to platform used and divided by diagnostic subgroup.

	All (*n* = 30)		N-P Cognitive Impairment (*n* = 10)		AD (*n* = 10)		FTD (*n*= 10)	
	Median	IQR	Median	IQR	Median	IQR	Median	IQR
**Age (years)**	61	54–66	51	43–56	60	56–66	67	63–68
**MMSE (score)**	27	23–28	29	27–30	25	24–27	23	19–27
**Disease** **duration (months)**	24	13–45	29	12–57	14	11–38	24	18–43
**NfL CSF (pg/mL)**								
**Ella^TM^**	879.0	510.0–3641.0	411.0	286.0–510.0	879.0	690.0–1166.0	6235.5	3641.0–7309.0
**Lumipulse^TM^**	735.5	365.0–2897.0	293.5	209.0–365.0	735.5	648.0–859.0	4785.0	2897.0–6443.0
**NfL serum (pg/mL)**								
**Ella^TM^**	21.00	13.30–58.40	11.15	7.69–17.40	21.60	15.80–27.70	109.00	62.60–137.00
**Lumipulse^TM^**	21.26	15.54–43.69	14.64	13.78–16.53	20.53	16.73–25.55	63.82	43.69–91.65

Notes: AD, Alzheimer’s Dementia; CSF, cerebrospinal fluid; FTD, Frontotemporal Dementia; N-P, non-progressive.

**Table 2:** Spearman’s correlation between neurofilament light chain (NfL) in cerebrospinal fluid (CSF) and serum measured with Ella™ and Lumipulse™.

Ella™ Versus Lumipulse™		
	**r**	**95% CI**
**NfL CSF**	0.976	0.950, 0.989
**NfL serum**	0.923	0.842, 0.964

**Table 3:** Passing–Bablok regression between Ella™ and Lumipulse™ neurofilament light chain (NfL) concentrations in cerebrospinal fluid (CSF) and serum. Values are intercept and slope with 95% confidence intervals (CI).

Parameter	Estimate	95% CI
**NfL CSF**		
**Intercept**	−45.38	−118.58, 29.42
**Slope**	0.8261	0.7038, 0.9289
**NfL serum**		
**Intercept**	7.543	5.828, 8.935
**Slope**	0.5775	0.5372, 0.6254

## Data Availability

The data presented in this study are available on request from the corresponding author due to privacy restriction reasons.

## Supplementary Materials

The following supporting information can be downloaded at https://www.mdpi.com/article/10.3390/diagnostics14212408/s1, Figure S1: Scatterplot matrix for neurofilament light chain (NfL) in serum and cerebrospinal fluid (CSF) concentrations using Ella^TM^ and Lumipulse^TM^; Table S1: Main characteristics of Lumipulse™ G NfL CSF and Blood and Ella Simple PlexTM Human NF-L Cartridge; Table S2: Median and interquartile range (IQR) concentrations of age, mini-mental state examination (MMSE) score, and neurofilament light chain (NfL) according to platform used and divided by sex; Table S3: Linear regression analyses of neurofilament light chain (NfL) in cerebrospinal fluid (CSF) and serum measured with Ella™ and Lumipulse™. We reported two statistical models: crude and adjusted-by-age models. Values are beta coefficients (β) with 95% confidence intervals (CIs).

## References

[1] Bomont P. (2021). The dazzling rise of neurofilaments: Physiological functions and roles as biomarkers. Curr. Opin. Cell Biol..

[2] Bridel C., van Wieringen W.N., Zetterberg H., Tijms B.M., Teunissen C.E., the NFL Group (2019). Diagnostic Value of Cerebrospinal Fluid Neurofilament Light Protein in Neurology: A Systematic Review and Meta-analysis. JAMA Neurol..

[3] Barro C., Chitnis T., Weiner H.L. (2020). Blood neurofilament light: A critical review of its application to neurologic disease. Ann. Clin. Transl. Neurol..

[4] Khalil M., Teunissen C.E., Otto M., Piehl F., Sormani M.P., Gattringer T., Barro C., Kappos L., Comabella M., Fazekas F. (2018). Neurofilaments as biomarkers in neurological disorders. Nat. Rev. Neurol..

[5] Yuan A., Nixon R.A. (2021). Neurofilament proteins as biomarkers to monitor neurological diseases and the efficacy of therapies. Front. Neurosci..

[6] Kalm M., Bostrom M., Sandelius A., Eriksson Y., Ek C.J., Blennow K., Bjork-Eriksson T., Zetterberg H. (2017). Serum concentrations of the axonal injury marker neurofilament light protein are not influenced by blood-brain barrier permeability. Brain Res..

[7] Uher T., McComb M., Galkin S., Srpova B., Oechtering J., Barro C., Tyblova M., Bergsland N., Krasensky J., Dwyer M. (2021). Neurofilament levels are associated with blood-brain barrier integrity, lymphocyte extravasation, and risk factors following the first demyelinating event in multiple sclerosis. Mult. Scler..

[8] Abu-Rumeileh S., Abdelhak A., Foschi M., D’Anna L., Russo M., Steinacker P., Kuhle J., Tumani H., Blennow K., Otto M. (2023). The multifaceted role of neurofilament light chain protein in non-primary neurological diseases. Brain.

[9] Lopes das Neves P., Duraes J., Silva-Spinola A., Lima M., Leitao M.J., Tabuas-Pereira M., Santana I., Baldeiras I. (2023). Serum Neurofilament Light Chain in the Diagnostic Evaluation of Patients with Cognitive Symptoms in the Neurological Consultation of a Tertiary Center. J. Alzheimer’s Dis..

[10] Notzel M., Werder L.I., Ziemssen T., Akgun K. (2022). Ella versus Simoa Serum Neurofilament Assessment to Monitor Treatment Response in Highly Active Multiple Sclerosis Patients. Int. J. Mol. Sci..

[11] Vecchio D., Puricelli C., Malucchi S., Virgilio E., Martire S., Perga S., Passarelli F., Valentino P., Di Sapio A., Cantello R. (2024). Serum and cerebrospinal fluid neurofilament light chains measured by SIMOA, Ella, and Lumipulse in multiple sclerosis naive patients. Mult. Scler. Relat. Disord..

[12] Truffi M., Garofalo M., Ricciardi A., Cotta Ramusino M., Perini G., Scaranzin S., Gastaldi M., Albasini S., Costa A., Chiavetta V. (2023). Neurofilament-light chain quantification by Simoa and Ella in plasma from patients with dementia: A comparative study. Sci. Rep..

[13] Mersch G., Dauwe M., De Geyter K., Vandeponseele P., Le Bastard N., Vandenbroucke I. Analytical performance of the Lumipulse^®^ G NfL CSF. Proceedings of the AD/PD 2023.

[14] McKhann G.M., Knopman D.S., Chertkow H., Hyman B.T., Jack C.R., Kawas C.H., Klunk W.E., Koroshetz W.J., Manly J.J., Mayeux R. (2011). The diagnosis of dementia due to Alzheimer’s disease: Recommendations from the National Institute on Aging-Alzheimer’s Association workgroups on diagnostic guidelines for Alzheimer’s disease. Alzheimer’s Dement..

[15] Gorno-Tempini M.L., Hillis A.E., Weintraub S., Kertesz A., Mendez M., Cappa S.F., Ogar J.M., Rohrer J.D., Black S., Boeve B.F. (2011). Classification of primary progressive aphasia and its variants. Neurology.

[16] Rascovsky K., Hodges J.R., Knopman D., Mendez M.F., Kramer J.H., Neuhaus J., van Swieten J.C., Seelaar H., Dopper E.G., Onyike C.U. (2011). Sensitivity of revised diagnostic criteria for the behavioural variant of frontotemporal dementia. Brain.

[17] Winblad B., Palmer K., Kivipelto M., Jelic V., Fratiglioni L., Wahlund L.O., Nordberg A., Backman L., Albert M., Almkvist O. (2004). Mild cognitive impairment--beyond controversies, towards a consensus: Report of the International Working Group on Mild Cognitive Impairment. J. Intern. Med..

[18] American Psychiatric Association (2013). Diagnostic and Statistical Manual of Mental Disorders.

[19] Willemse E.A., Teunissen C.E. (2015). Biobanking of cerebrospinal fluid for biomarker analysis in neurological diseases. Biobanking in the 21st Century.

[20] Tondelli M., Salemme S., Vinceti G., Bedin R., Trenti T., Molinari M.A., Chiari A., Zamboni G. (2022). Predictive value of phospho-tau/total-tau ratio in amyloid-negative Mild Cognitive Impairment. Neurosci. Lett..

[21] Urbano T., Chiari A., Malagoli C., Cherubini A., Bedin R., Costanzini S., Teggi S., Maffeis G., Vinceti M., Filippini T. (2023). Particulate matter exposure from motorized traffic and risk of conversion from mild cognitive impairment to dementia: An Italian prospective cohort study. Environ. Res..

[22] Vinceti M., Urbano T., Chiari A., Filippini T., Wise L.A., Tondelli M., Michalke B., Shimizu M., Saito Y. (2023). Selenoprotein P concentrations and risk of progression from mild cognitive impairment to dementia. Sci. Rep..

[23] Martinelli I., Zucchi E., Simonini C., Gianferrari G., Bedin R., Biral C., Ghezzi A., Fini N., Carra S., Mandrioli J. (2024). SerpinA1 levels in amyotrophic lateral sclerosis patients: An exploratory study. Eur. J. Neurol..

[24] Vinceti M., Urbano T., Filippini T., Bedin R., Simonini C., Sorarù G., Trojsi F., Michalke B., Mandrioli J. (2024). Changes in Cerebrospinal Fluid Concentrations of Selenium Species Induced by Tofersen Administration in Subjects with Amyotrophic Lateral Sclerosis Carrying SOD1 Gene Mutations. Biol. Trace Elem. Res..

[25] The MathWorks Inc (2022). MATLAB, R2022a.

[26] Urbano T., Filippini T., Malavolti M., Fustinoni S., Michalke B., Wise L.A., Vinceti M. (2023). Adherence to the Mediterranean-DASH Intervention for Neurodegenerative Delay (MIND) diet and exposure to selenium species: A cross-sectional study. Nutr. Res..

[27] Urbano T., Verzelloni P., Malavolti M., Sucato S., Polledri E., Agnoli C., Sieri S., Natalini N., Marchesi C., Fustinoni S. (2023). Influence of dietary patterns on urinary excretion of cadmium in an Italian population: A cross-sectional study. J. Trace Elem. Med. Biol..

[28] Alcolea D., Beeri M.S., Rojas J.C., Gardner R.C., Lleo A. (2023). Blood Biomarkers in Neurodegenerative Diseases: Implications for the Clinical Neurologist. Neurology.

[29] Arslan B., Zetterberg H. (2023). Neurofilament light chain as neuronal injury marker—what is needed to facilitate implementation in clinical laboratory practice?. Clin. Chem. Lab. Med..

[30] Bayoumy S., Verberk I.M.W., Vermunt L., Willemse E., den Dulk B., van der Ploeg A.T., Pajkrt D., Nitz E., van den Hout J.M.P., van der Post J. (2024). Neurofilament light protein as a biomarker for spinal muscular atrophy: A review and reference ranges. Clin. Chem. Lab. Med..

[31] Benatar M., Ostrow L.W., Lewcock J.W., Bennett F., Shefner J., Bowser R., Larkin P., Bruijn L., Wuu J. (2024). Biomarker Qualification for Neurofilament Light Chain in Amyotrophic Lateral Sclerosis: Theory and Practice. Ann. Neurol..

[32] Gallingani C., Carbone C., Tondelli M., Zamboni G. (2024). Neurofilaments Light Chain in Neurodegenerative Dementias: A Review of Imaging Correlates. Brain Sci..

[33] Gauthier A., Viel S., Perret M., Brocard G., Casey R., Lombard C., Laurent-Chabalier S., Debouverie M., Edan G., Vukusic S. (2021). Comparison of Simoa(TM) and Ella(TM) to assess serum neurofilament-light chain in multiple sclerosis. Ann. Clin. Transl. Neurol..

[34] Giuffre G.M., Quaranta D., Costantini E.M., Citro S., Martellacci N., De Ninno G., Vita M.G., Guglielmi V., Rossini P.M., Calabresi P. (2023). Cerebrospinal fluid neurofilament light chain and total-tau as biomarkers of neurodegeneration in Alzheimer’s disease and frontotemporal dementia. Neurobiol. Dis..

[35] Walia N., Eratne D., Loi S.M., Farrand S., Li Q.X., Malpas C.B., Varghese S., Walterfang M., Evans A.H., Parker S. (2023). Cerebrospinal fluid neurofilament light and cerebral atrophy in younger-onset dementia and primary psychiatric disorders. Intern. Med. J..

[36] Bolsewig K., Hok A.H.Y.S., Sepe F.N., Boonkamp L., Jacobs D., Bellomo G., Paoletti F.P., Vanmechelen E., Teunissen C.E., Parnetti L. (2022). A Combination of Neurofilament Light, Glial Fibrillary Acidic Protein, and Neuronal Pentraxin-2 Discriminates Between Frontotemporal Dementia and Other Dementias. J. Alzheimer’s Dis..

[37] Saji N., Murotani K., Sato N., Tsuduki T., Hisada T., Shinohara M., Sugimoto T., Niida S., Toba K., Sakurai T. (2022). Relationship Between Plasma Neurofilament Light Chain, Gut Microbiota, and Dementia: A Cross-Sectional Study. J. Alzheimer’s Dis..

[38] Fang T., Dai Y., Hu X., Xu Y., Qiao J. (2024). Evaluation of serum neurofilament light chain and glial fibrillary acidic protein in the diagnosis of Alzheimer’s disease. Front. Neurol..

[39] Andersson E., Janelidze S., Lampinen B., Nilsson M., Leuzy A., Stomrud E., Blennow K., Zetterberg H., Hansson O. (2020). Blood and cerebrospinal fluid neurofilament light differentially detect neurodegeneration in early Alzheimer’s disease. Neurobiol. Aging.

[40] Kuhle J., Barro C., Andreasson U., Derfuss T., Lindberg R., Sandelius A., Liman V., Norgren N., Blennow K., Zetterberg H. (2016). Comparison of three analytical platforms for quantification of the neurofilament light chain in blood samples: ELISA, electrochemiluminescence immunoassay and Simoa. Clin. Chem. Lab. Med..

[41] Alagaratnam J., von Widekind S., De Francesco D., Underwood J., Edison P., Winston A., Zetterberg H., Fidler S. (2021). Correlation between CSF and blood neurofilament light chain protein: A systematic review and meta-analysis. BMJ Neurol. Open.

[42] Koini M., Pirpamer L., Hofer E., Buchmann A., Pinter D., Ropele S., Enzinger C., Benkert P., Leppert D., Kuhle J. (2021). Factors influencing serum neurofilament light chain levels in normal aging. Aging (Albany NY).

[43] Pifferi F., Laurent B., Plourde M. (2021). Lipid transport and metabolism at the blood-brain interface: Implications in health and disease. Front. Physiol..

[44] Luigetti M., Primiano G., Basile V., Vitali F., Pignalosa S., Romano A., Sabino A., Marino M., Di Santo R., Ciasca G. (2024). Serum Neurofilament and Free Light Chain Levels in Patients Undergoing Treatment for Chronic Inflammatory Demyelinating Polyneuropathy. Int. J. Mol. Sci..

[45] Khalil M., Pirpamer L., Hofer E., Voortman M.M., Barro C., Leppert D., Benkert P., Ropele S., Enzinger C., Fazekas F. (2020). Serum neurofilament light levels in normal aging and their association with morphologic brain changes. Nat. Commun..

