# Correction: Urbano et al. Comparison of Serum and Cerebrospinal Fluid Neurofilament Light Chain Concentrations Measured by Ella™ and Lumipulse™ in Patients with Cognitive Impairment. *Diagnostics* 2024, *14*, 2408

**DOI:** 10.3390/diagnostics16081177

**Published:** 2026-04-16

**Authors:** Teresa Urbano, Riccardo Maramotti, Manuela Tondelli, Chiara Gallingani, Chiara Carbone, Najara Iacovino, Giulia Vinceti, Giovanna Zamboni, Annalisa Chiari, Roberta Bedin

**Affiliations:** 1 Neuroimmunology Laboratory, Department of Biomedical, Metabolic and Neural Sciences, University of Modena and Reggio Emilia, Baggiovara Hospital, 41126 Modena, Italy; teresa.urbano@unimore.it (T.U.); roberta.bedin@unimore.it (R.B.); 2 Environmental, Genetic and Nutritional Epidemiology Research Center (CREAGEN), Department of Biomedical, Metabolic and Neural Sciences, University of Modena and Reggio Emilia, 41125 Modena, Italy; 3 Department of Biomedical, Metabolic and Neural Sciences, University of Modena and Reggio Emilia, 41125 Modena, Italy; riccardo.maramotti@unimore.it (R.M.); gallinganichiara@gmail.com (C.G.); chiara.carbone@unimore.it (C.C.); najara.iacovino@unimore.it (N.I.); giovanna.zamboni@unimore.it (G.Z.); 4 Department of Physics, Informatics and Mathematics, University of Modena and Reggio Emilia, 41125 Modena, Italy; 5 Department of Mathematics and Computer Science, University of Ferrara, 44121 Ferrara, Italy; 6 Neurology Unit, Baggiovara Hospital, 41126 Modena, Italy; gvinceti@gmail.com (G.V.); chiari.annalisa@aou.mo.it (A.C.)

In the original publication [1], there was a transcription error in the mathematical expressions of the conversion formulae between the Lumipulse™ and Ella™ assays derived from the Passing–Bablok regression model. A correction has been made to Section 3 *Results*, Paragraphs 7 and 8 with equations, as follows:

When only Ella™ is available, the formulae used to obtain the corresponding value in Lumipulse™ (y) in relation to Ella™ (x) are

y(Lumipulse™) = 0.8261 × x(Ella™) − 45.38 (for CSF)

y(Lumipulse™) = 0.5775 × x(Ella™) + 7.543 (for serum)

On the other hand, when only Lumipulse™ (y) is available, the formulae used to obtain the corresponding Ella™ (x) value are

x(Ella™) = 1.211 × y(Lumipulse™) + 45.38 (for CSF)

x(Ella™) = 1.732 × y(Lumipulse™) − 7.543 (for serum)

The authors state that the scientific conclusions are unaffected. This correction was approved by the Academic Editor. The original publication has also been updated.
