# Supplementary material for: Comparison of Serum and Cerebrospinal Fluid Neurofilament Light Chain Concentrations Measured by Ella™ and Lumipulse™ in Patients with Cognitive Impairment
Source: Diagnostics (Basel). 2024 Oct 29;14(21):2408. doi: 10.3390/diagnostics14212408 (PMC11544876; doi:10.3390/diagnostics14212408)

Supplementary Material for "Comparison of serum and cerebrospinal fluid neurofilament light chain concentrations measured by Ella™ and Lumipulse™ in patients with cognitive impairment"

Supplementary Tables

Table S1. Main characteristics of Lumipulse™ G NfL CSF and Blood, and Ella Simple Plex™ Human NF-L Cartridge.

Features	Lumipulse™ G NfL CSF and blood	Ella Simple Plex™ Human NF-L Cartridge
**Assay design**	Specific two-step sandwich CLEIA	Specific two-step fluorescence-based immunoassay
**Sample type**	CSF, serum	CSF, serum
**Sample volume**	CSF: 60 μL<br>Serum: 100 μL	CSF and serum: 25 μL
**Measuring range**	CSF: 4.0-50000.0 pg/mL<br>Serum: 2.0-5000.0 pg/mL	2.7-10290.0 pg/mL
**Limit of detection (LOD)**	CSF: 4 pg/mL<br>Serum: 2.99 pg/mL	1.09 pg/mL
**Limit of quantification (LOQ)**	CSF: 6 pg/mL<br>Serum: 3.25 pg/mL	2.70 pg/mL
**Imprecision**	CSF: 2.2-7.0%<br>Serum: 3.1-4.3%	Not provided
**Calibrators, calibration points and calibration curve stability**	Ready-to-use (around 800, 4000, and 20000 pg/mL), 5 points, 30 days	Cartridge calibrator, 12 points, factory-generated calibration curve

Table S2. Median and interquartile range (IQR) concentrations of age, mini-mental state examination (MMSE) score, and neurofilaments light chain (NfL) according to platform used and divided by sex.

	All (n=30)		Males (n=8)		Females (n=22)	
	Median	IQR	Median	IQR	Median	IQR
Age (years)	61	54-66	62	58-66	59	54-66
MMSE (score)	27	23-28	24	20-28	27	23-28
Disease duration (months)	24	13-45	17	13-41	26	13-46
**NfL CSF** (pg/mL)						
Ella™	879.0	510.0-3641.0	1217.0	614.0-3515.5	761.5	438.0-3764.0
Lumipulse™	735.5	365.0-2897.0	1005.0	526.5-2875.0	686.5	332.0-4369.0
**NfL serum** (pg/mL)						
Ella™	21.00	13.30-58.40	23.00	13.30-57.60	19.75	11.50-81.80
Lumipulse™	21.26	15.54-43.69	30.21	17.63-42.44	19.26	14.66-52.88

Table S3. Linear regression analyses of neurofilament light chain (NfL) in cerebrospinal fluid (CSF) and serum measured with Ella™ and Lumipulse™. We reported two statistical models: crude and adjusted by age. Values are beta coefficients (β) with 95% confidence intervals (CI).

**Ella™ versus Lumipulse™**	**Crude model**	**Adjusted for age**
	β (95% CI)	β (95% CI)
**NfL CSF**	0.77 (0.68-0.86)	0.79 (0.68-0.90)
**NfL serum**	0.64 (0.55-0.73)	0.65 (0.54-0.77)

Supplementary Figures

Figure S1. Scatterplot matrix for neurofilament light chain (NfL) in serum and cerebrospinal fluid (CSF) concentrations using Ella™ and Lumipulse™.

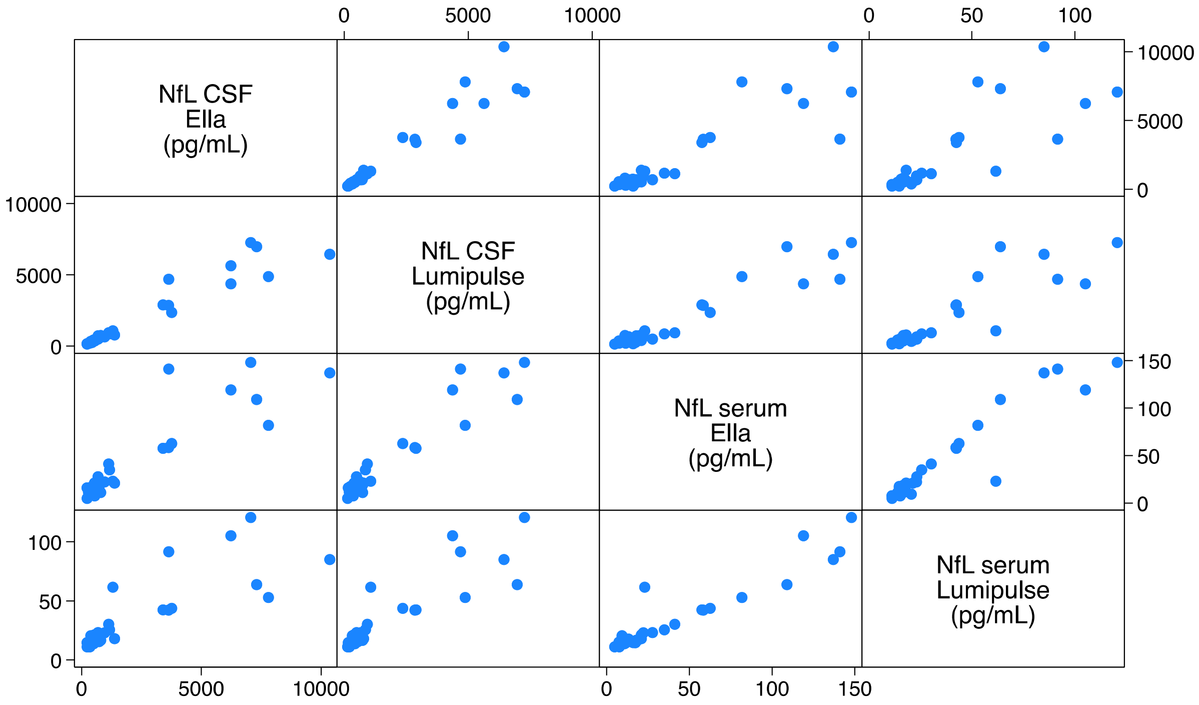

Supplement: Supplementary file 1 [file diagnostics-14-02408-s001.zip › diagnostics-3229395-supplementary.pdf]
